# Hemostasis system condition in infectious complication development in severe burned patients

**DOI:** 10.1186/cc11044

**Published:** 2012-03-20

**Authors:** M Presnyakova, V Kuznetsova

**Affiliations:** 1 Nizhny Novgorod Research Institute of Traumatology and Orthopedics, Nyzhny Novgorod, Russia

## Introduction

Over the period of the history of combustiology one of the main problems for treatment of patients with burns is infection, both local - bacterial pneumonia - and generalized - sepsis - characterized by extremely severe course, complex diagnostics and high lethality rate. However, the role of hemostasis disorders in infectious complication development in severe burned patients is taken into consideration insufficiently. The aim of the study is to reveal the most relevant hemostasis system changes in sepsis and pneumonia in patients with serious heat injury in an acute period of burn disease.

## Methods

Hemostasis and biochemical blood parameters were studied in 169 patients with over 20% of the body burned, from the first to 12th days after burn. Sepsis developed in 33 patients, 69 patients had pneumonia, and in 67 patients there were no complications of sepsis and pneumonia. Infectious septic complications were diagnosed in the clinic on the basis of clinical and laboratory findings, as well as confirmed by morphological studies in casualties (44 from 102 patients). Diagnosis of disseminated intravascular coagulation (DIC) syndrome was made based on standard criteria.

## Results

The analysis of findings showed both sepsis and pneumonia development in an acute period of burn disease to be accompanied by disorders of anticoagulant, fibrinolytic and procoagulant parts of the hemostasis system typical for DIC syndrome. The changes of hemostasis system indices were not only the characteristic of infection in burned patients but they preceded the diagnosis of sepsis and pneumonia in the clinic on average by 2 to 4 days. In patients with pneumonia, relevant and statistically significant were the activity changes of XIIa-dependent fibrinolysis, from the second to sixth days. And on the third to seventh days there was reliable pneumonia development with decreased activity of antithrombin III. In patients with sepsis were revealed changes of XIIa-dependent fibrinolysis activity - from the third to seventh days - and antithrombin III activity - from the third to the sixth days.

## Conclusion

The development of both local and generalized infection in an acute period of burn disease occurs against the background of DIC syndrome induced by a serious heat injury. The indices of hemostasis system can be included into a complex of clinic and laboratory studies aimed at detecting infection and early intensive etiopathological therapy.

